# Do Cultivar, Geographical Location and Crop Season Influence Phenolic Profile of Walnut Leaves?

**DOI:** 10.3390/molecules13061321

**Published:** 2008-06-12

**Authors:** Joana S. Amaral, Patrícia Valentão, Paula B. Andrade, Rui C. Martins, Rosa M. Seabra

**Affiliations:** 1 REQUIMTE, Serviço de Farmacognosia, Faculdade de Farmácia, Universidade do Porto, R. Aníbal Cunha, 164, 4099-030 Porto, Portugal; E-mails: valentao@ff.up.pt, pandrade@ff.up.pt, rseabra@ff.up.pt; 2 Escola Superior de Tecnologia e de Gestão, Instituto Politécnico de Bragança, Quinta de Sta. Apolónia, Apartado 134, 5301-857 Bragança, Portugal; 3 Centre for Biological Engineering, University of Minho, Campus of Gualtar, 4710-057 Braga, Portugal; E-mail: rui.martins@deb.uminho.pt

**Keywords:** Walnut leaves, *Juglans regia* L., phenolic compounds, flavonoids, HPLC-DAD, multivariate analysis

## Abstract

Walnut leaves from nine different cultivars (Arco, Franquette, Hartley, Lara, Marbot, Mayette, Meylannaise, Parisienne and Rego) were studied for their phenolic compounds. Samples were harvested along three consecutive years, at two different geographical locations, in order to evaluate if significant differences in the phenolics composition can be related with genetic, climatic or geographical factors. Nine compounds (3-caffeoylquinic, 3-*p*-coumaroylquinic and 4-*p*-coumaroylquinic acids, quercetin 3-galactoside, quercetin 3-arabinoside, quercetin 3-xyloside, quercetin 3-rhamnoside, a quercetin 3-pentoside derivative and a kaempferol 3-pentoside derivative) were quantified using an HPLC-DAD methodology. The qualitative profiles were identical for all samples, but differences were observed in terms of individual compounds’ contents. Multivariate statistical analysis was carried out, showing that significant differences exist among production years, which can be related to climatic reasons.

## Introduction

Walnut tree (*Juglans regia* L.), is native to Eastern Europe and North Asia, but is also found throughout North, Central and South America. The tree has great socio-economic importance being frequently cultivated in temperate zones of the world mainly because of its edible seed, whose oil is rich in unsaturated fatty acids, phytosterols and tocopherols [1, 2] and whose consumption has recently been related to health benefits [3,4,5]. Moreover, its non-edible parts such as leaves, husks and wood also find broad application; its use is reported to flavour liqueurs [6,7,8], in cosmetics [9], in dyes [10], furniture and in traditional medicine [11].

Since ancient times, traditional medicinal plants have played important roles in public health, being a source of health care and disease prevention, especially in non-developed and developing countries. However, nowadays industrially-prepared herbal products are gaining market importance in the so called developed countries. In Europe, the use of plants with pharmaceutical properties is receiving an increased interest among the general public, with several herbal products being available and widely used in all Member States of the European Union [12].

Walnut leaf has been widely used in folk medicine for treatment of venous insufficiency and haemorrhoidal symptomatology, and for its antidiarrheic, antihelmintic, depurative and astringent properties [11,13,14]. Keratolytic, antifungal, hypoglycaemic, hypotensive, anti-scrofulous and sedative activities have also been described [15,16]. Several of these traditionally attributed actions may be due to tannins known to occur in these leaves [17], but also to several phenolic compounds, namely to flavonoids and phenolic acids. In fact, several pharmacological effects have been ascribed to flavonoids, such as anti-inflammatory, antihepatotoxic, antitumor, antimicrobial, antiviral, enzyme inhibiting and as having central vascular effects [18]. This herbal drug was officially listed in the 10th edition of the French Pharmacopoeia [19] where it was proposed that its chemical quality control be accomplished by TLC detection of quercetin 3-galactoside and quercetin 3-rhamnoside. This test is obviously insufficient if we consider that this set of compounds is almost ubiquitous. In a previous study [20], a deeper study of the phenolic composition of walnut leaves was made using HPLC/DAD/MS/MS – ESI, and a useful methodology for routine quality control of this commercial medicinal plant, based on HPLC-DAD quantification of major phenolics, was developed. In that study, samples from several cultivars, originating from a same orchard and from a same year of production, were analysed for their variation from May to September. However it is a known fact that the active chemical constituents of medicinal plants can be affected by numerous factors, including genetic differences, geographical locations, climatic conditions and agricultural practices [21]. Despite this general concept, which is sometimes used to reject herbal medicines, the detailed study of the magnitude and direction of the influence of such factors is very scarce, making standardisation of traditional drugs a difficult task.

Having in mind the lack of information in this area, namely with regards to quality control of walnut leaves, this study was designed to characterize the differences in phenolics among different cultivars, grown in two geographical origins during a period of three consecutive years.

## Results and Discussion

A study concerning the identification of the phenolic compounds by HPLC/DAD/ESI-MS/MS in walnut leaf has been previously performed [20]. In that study, seven phenolic compounds: 3-caffeoyl-quinic, 3-*p*-coumaroylquinic and 4-*p*-coumaroylquinic acids, quercetin 3-galactoside, quercetin 3-arabinoside, quercetin 3-xyloside, quercetin 3-rhamnoside, and two partially identified compounds: a quercetin 3-pentoside derivative and a kaempferol 3-pentoside derivative, were identified.

**Table 1:** Phenolic composition^a ^(g/kg, dry basis) of the studied cultivars, by year of production and geographical locality.

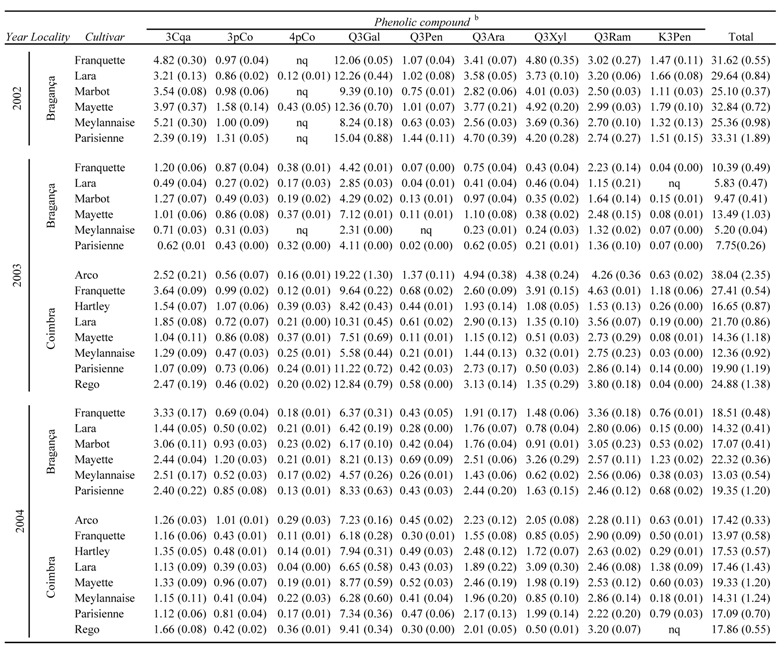

**Figure 1:**
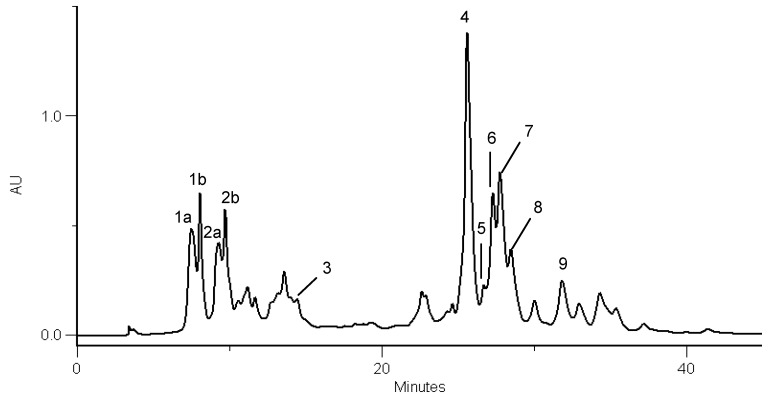
HPLC/DAD walnut leaf phenolic profile (Parisienne cultivar, sampled in Bragança, 2002). Detection at 320 nm. Peaks, 1a and 1b: 3-caffeoylquinic acid isomers; 2a and 2b: 3-*p-*coumaroylquinic acid isomers; 3: *4-p-*coumaroylquinic acid; 4: quercetin 3-galactoside; 5: quercetin 3-pentoside derivative; 6: quercetin 3-arabinoside; 7: quercetin 3-xyloside; 8: quercetin 3-rhamnoside; 9: kaempferol 3-pentoside derivative.

^a^ Values are expressed as mean (standard deviation) of three determinations for each sample. nq: not quantified. ^b^ Identity of compounds as in Figure 1.

The average content and corresponding standard deviation for each phenolic compound of the studied cultivars, by year of production and geographical location, is shown in Table 1 and Figure 1 shows the phenolic fingerprint built with such values. Table 1 also displays a column with the sum of the identified phenolics in each sample.

**Figure 2:**
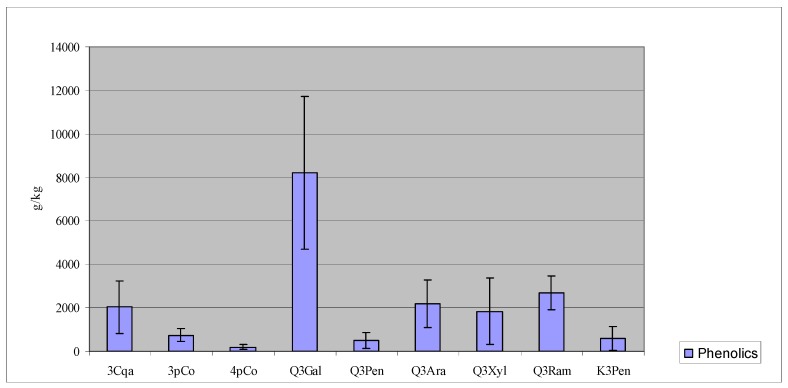
Walnut leaf phenolic fingerprint. Results are the mean of all studied samples; error bars represent the standard deviation.

The first striking conclusion that can be drawn on observing that column is the wide variation from a minimum of 5.20 g/kg to a maximum of 38.04 g/kg, which represents a seven fold range. Given this scenario, a deeper analysis of the individual values became necessary in order to check for the factors that caused the mentioned variation and if such variation affected or not the phenolic fingerprint presented in Figure 2.

Consequently, the results obtained from the analysis of the 34 samples performed in triplicate (102 total data points) were statistically analysed. In a first approach, a correlation analysis was performed. High correlations were found between all combinations of the following phenolic compounds: quercetin 3-galactoside, quercetin 3-pentoside, quercetin 3-arabinoside and quercetin 3-xyloside, since the obtained values of the Pearson correlation coefficient were from 0.8332 to 0.9186 and the values of the Spearman correlation coefficient were from 0.9186 to 0.9395.

**Figure 3:**
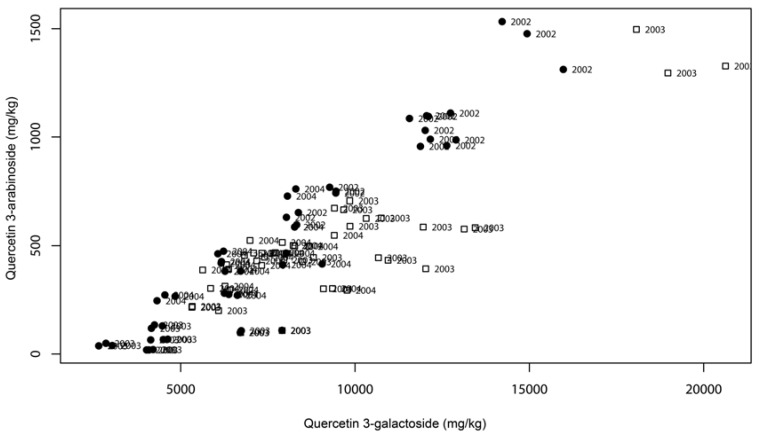
Walnut leaves correlation plot: quercetin 3-arabinoside *vs* quercetin 3-galactoside. Symbols: ●: samples from Bragança; □: samples from Coimbra.

Figure 3 shows the positive correlation found between quercetin 3-arabinoside and quercetin 3-galactoside, showing that the samples containing higher levels of quercetin 3-arabinoside were also higher in quercetin 3-galactoside. Identical graphics were obtained for all combinations of the four compounds (data not shown). These positive correlations appear to be independent of the year and geographical location, for the generality of the studied cultivars.

Quercetin 3-galactoside presented the largest variation between the minimum and maximum values over the years 2002 to 2004 (2.3 to 19.2 g/kg); quercetin 3-arabinoside and quercetin 3-xyloside variations were from 0.2 to 3.8 g/kg and from 0.2 to 4.9 g/kg, respectively; quercetin 3-pentoside presented a variation from 0.02 to 1.4 g/kg. These four compounds were also strongly correlated with the total amount of phenolics, pointing that the large variability of this group of compounds strongly influenced the total amount of phenolics in the analyzed samples. This means that the graphic displayed on Figure 2 represents, with a significant level of confidence, the phenolic fingerprint of walnut leaves because although total amounts of phenolics can vary widely, the proportions among the most of them is reasonably constant. Figure 3 also shows that significant differences seem to exist among the three production years; on the contrary, significant differences between the two geographical locations were not evident.

The differences between the phenolic content over the three production years is sustained by the analysis of variance (*F*-test) *p*-values presented in Table 2, since significant differences (*p*<0.001) exist for all phenolic compounds, with the exception of kaempferol 3-pentoside. Significant differences were also found among samples of different cultivars and among samples from different geographical localities (Table 2), showing that at least one sample is different from the others. The analysis of variance also suggested the existence of outlier samples in terms of cultivar and geographical location. In the Tukey multicomparison test, results suggested, at a 95% confidence level, that the cultivar Arco (Coimbra, 2003) was an atypical sample from all the observations in terms of phenolic profile. The same test also showed that the cultivar Parisienne (Bragança, 2002) is also distinguishable from the remaining cultivars from Bragança during the years 2002, 2003 and 2004.

**Table 2:** Analysis of variance (*F*-test) results.

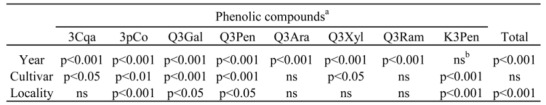

^a ^3Cqa: 3-caffeoylquinic acid; 3pCo: 3-*p-*coumaroylquinic acid; Q3gal: quercetin 3-galactoside; Q3Pen: quercetin 3-pentoside derivative; Q3Ara: quercetin 3-arabinoside; Q3Xyl: quercetin 3-xyloside; Q3Ram: quercetin 3-rhamnoside; K3Pen: kaempferol 3-pentoside derivative. ^b^ ns: statistically non significative.

**Figure 4:**
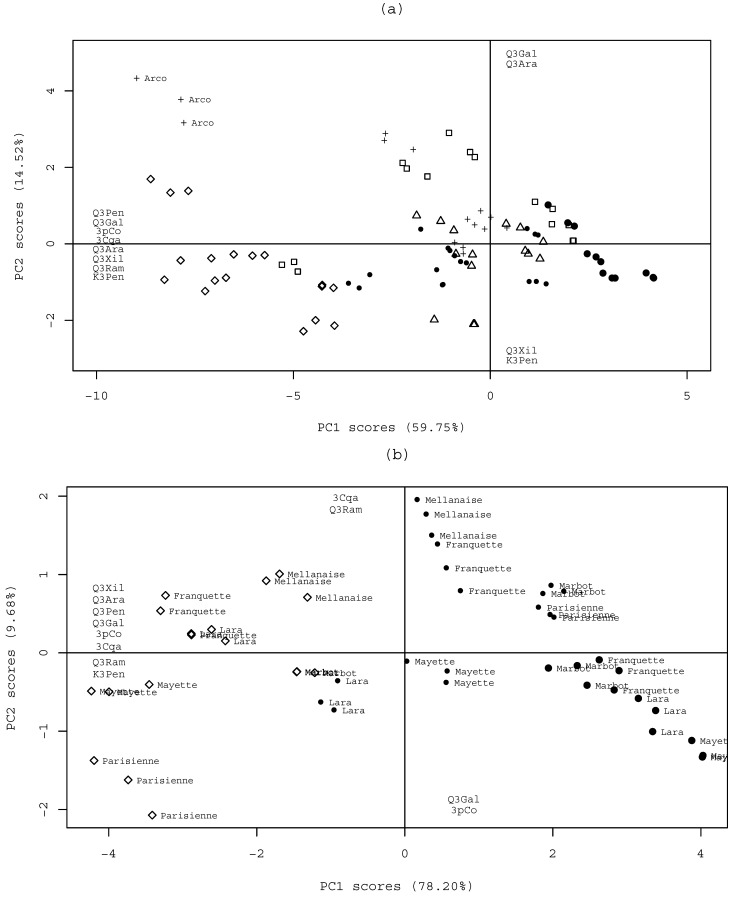
Principal Component Analysis Scores: (a) PC scores of common cultivars of Bragança and Coimbra from 2003 and 2004 production years, with the scores projection of Bragança 2002 data and the non-common cultivars from Coimbra data (Arco, Hartley and Rego); (b) PC scores of Bragança 2002, 2003 and 2004 data. Symbols: ◊: Bragança 2002; ●: Bragança 2003; •: Bragança 2004; □: Coimbra 2003; ∆: Coimbra 2004; +: non-common cultivars from Coimbra (Arco, Hartley and Rego).

The significant differences were thereafter analysed by PCA to identify tendencies in the phenolic profile pattern. Initially, a principal components analysis score plot was obtained with a linear model of all the phenolic constituents (with the exception of 4-*p*-coumaroylquimic acid, due to excessive missing data; see Table 1), using only the data regarding the cultivars common to Bragança and Coimbra (Franquette, Lara, Mayette, Meylannaise and Parisienne) and concerning the common production years (2003 and 2004) (see Table 1). As no significant differences among cultivars and geographical locations were evident, the samples from the 2002 year crop from Bragança and the non-common cultivars from Coimbra were also added to the analyses. The global picture of major differences is shown in the obtained principal components analysis score plot (Figure 4a). Two main PCs accounted for 74.3% of the total variability, PC1 (59.8%) and PC2 (14.5%). PC1 loadings express the majority of phenolic compounds content, where negative scores represent samples with high phenolic content. PC2 presents the inverse relationship between the content of quercetin 3-galactoside and quercetin 3-arabinoside versus quercetin 3-xyloside and kaempferol 3-pentoside, where data suggests that samples rich in the first two compounds are poor in the second group of compounds. Significant differences among geographical locations and cultivars are not evident in terms of the phenolic content in the Gabriel plot of Figure 4a.

Although some samples of the 2003 year crop from Bragança were clearly distinct from the Coimbra ones, in general, the samples from the two geographical localities were similar in terms of phenolic composition. Nevertheless, the cultivar Arco (Coimbra 2003) has clearly shown to be an atypical sample, presenting significantly higher phenolic contents than the remaining of the 2003 and 2004 samples, and confirming the same observation during the analysis of variance.

Figure 4a also allows observing that the predicted scores of the phenolic profile of Bragança samples from the year 2002 (represented with the symbol ◊) are clearly separated, exhibiting a significantly higher phenolic content than the other analysed samples. This fact, lead us to perform another PCA with only the data from the Bragança samples (excluding the information from Coimbra, i.e., excluding the data variance associated with the geographical location factor), aiming to distinguish patterns during the 3 years of study. Figure 4b shows the plot obtained, were two main PCs accounted for 87.88% of total variance. The variables important to explain differences are plotted in the correspondent axes. PC1 represents 78.28% of the information of the data, separating the 3 production years, reflecting the higher and lower phenolics contents found in 2002 and 2003, respectively (Figure 4b). Furthermore, it is observable that the Parisienne cultivar in 2002 is distinguishable from the remaining of the 2002 data group, confirming the result obtained during variance analysis. Moreover, it is observable that 2003 and 2004 year crops are mainly separated by the contents of 3-caffeoylquinic acid and quercetin 3-rhamnoside (higher in 2003) and by the contents of 3-*p*-coumaroylquinic acid and quercetin 3-galactoside (higher in 2004).

The pointed differences can possibly be related to the climatic differences among the years in question. In Portugal, 2004 was characterized by the lowest rainfall since 1931 (rainfall < 401 mm) [22]. During this year, both Bragança and Coimbra were considered to be under severe drought conditions [22]. Furthermore, June (which corresponds to month previous to the collection of the walnut leaves), was exceptionally dry, with high temperatures.

Comparing the years 2002 and 2003, between January and June, several differences in terms of rainfall were observed (data not shown). In terms of temperatures, the year 2003 was characterized by a very hot summer, the second hotter since 1931 [23], although this probably did not affect the samples of the 2003 production year since the samples were collected in July and the heat wave was observed between 29th July and 15th August. Although it is difficult to draw conclusions on how the climate conditions may affect the phenolic profile of walnut leaves, the obtained results suggest that the content of the phenolic compounds in walnut leaves can be influenced by climatic conditions since the differences observed between years of production were based on the same cultivars (grown in the same experimental field and under the same agricultural practices). A deeper study concerning climatic data in-situ and also the performance of the record of the agricultural practices is therefore recommended to withdraw deeper conclusions on the walnut leaf phenolic profile variation.

## Conclusions

The studied walnut leaf samples presented a common qualitative profile comprising nine phenolic compounds. Although multivariate statistical analysis showed that, in general, the samples of different cultivars and from different geographical locations were identical in terms of their phenolics composition and contents, significant differences were found among production years. These differences can possibly be related to the natural climatic differences that occur over the years. A deeper study with the recording of climatic data *in-situ* and agricultural practices, during a longer period of time is therefore recommended to extract deeper conclusions about the walnut leaf phenolic profile variation, and to confirm if the pointed correlation between the referred group of compounds is in fact characteristic of the species. If these preliminary conclusions are confirmed, it seems advisable to apply a routine methodology in the quality control of this product, since this kind of compounds have been associated with several beneficial pharmacological effects and different amounts can exist in the commercial plants.

## Experimental

### Samples

Studies were carried out on walnut leaves from nine cultivars (Arco, Franquette, Hartley, Lara, Marbot, Mayette, Meylannaise, Parisienne and Rego). Fresh leaves of the different cultivars were collected in July in two geographical origins: Bragança, northeastern region of Portugal (6º46´W, 41º49´N, 670m elevation) during three consecutive years (2002, 2003 and 2004), and in Coimbra (8º37´W, 40º03´N, 10 m elevation), in the center region of Portugal in two years (2003 and 2004). Samples of cultivars Franquette, Lara, Mayette, Meylannaise and Parisienne were common for both the geographical origins; cv. Marbot only existed in Bragança, and cvs. Arco, Hartley and Rego only existed in Coimbra. The walnut trees cultivated in Bragança region were approximately eighteen years old and were planted with a density of 7 x 7m. The trees are pruned when necessary, receive organic fertilization but no phytosanitary treatments, the surrounding soil is tilled to control infestation and the trees are irrigated in summer (July and August). The Coimbra region walnut tree samples were obtained from younger trees, approximately ten years old, and planted with a density of 8 x 6m or 8 x 8m, depending on the cultivar. They were pruned when necessary, the soil received organic fertilization, phytosanitary treatments are applied between April and August if necessary, and they are irrigated at the end of spring and in summer (May to August). Samples of developed leaves were manually collected from the tip of the annual shoots inserted in the middle third of vegetative branches exposed to sunlight, dried in a stove at 30 ºC and stored in paper bags in order to protect them from light. Before phenolic extraction, each sample was powdered to pass through a 910 μm sieve.

### Reagents and Standards

The standards were purchased from Sigma (St. Louis, MO, USA) and Extrasynthese (Genay, France). Methanol and hydrochloric and formic acids were obtained from Merck (Darmstadt, Germany). The water was treated in a Milli-Q water purification system (Millipore, Bedford, MA, USA).

### Phenolic compounds extraction

The extraction of the phenolic compounds was achieved as previously reported [20]. Briefly, each sample (ca. 0.2 g) was mixed with acidified water (pH 2 with HCl) until complete extraction of phenolics (negative reaction to NaCl 20%) and filtered. The filtrate was passed trough an ISOLUTE NEC C18 column (International Sorbent Tecnology Ltd., Mid Glamorgan, UK) previously preconditioned with 60 mL of methanol, followed by 140 mL of water (pH 2 with HCl). The retained phenolic fraction was eluted with methanol (ca. 75 mL), evaporated to dryness under reduced pressure (40 ºC) and redissolved in methanol (3 mL).

### Equipment

The extracts were analysed on an analytical HPLC unit (Gilson), using a Spherisorb ODS2 column (250 x 4.6 mm, 5 μm particle size, Merck, Darmstadt, Germany). Elution was performed at a flow rate of 1 mL/min with water/formic acid (19:1) (solvent A) and methanol (solvent B) starting with 5% B and running a gradient to obtain 15% B at 3 min, 20% B at 5min, 25% B at 12 min, 30% B at 15 min, 40% B at 20 min, 45% B at 30 min, 50% B at 40 min, 70% B 45 min and 0% B at 46 min. The injection volume was 20 μL. Detection was achieved with a Gilson DAD and data were processed on a Unipoint system software (Gilson Medical Electronics, Villiers le Bel, France). Phenolic compounds quantification was achieved by the absorbance recorded in the chromatograms relative to external standards, with detection at 320 nm for phenolic acids and at 350 nm for flavonoids. 3-*O*-Caffeoylquinic acid was quantified as 5-*O*-caffeoylquinic acid, 3-*p*-coumaroylquinic and 4-*p*-coumaroylquinic acids were quantified as *p*-coumaric acid; the quercetin 3-pentoside derivative and quercetin 3-xyloside were quantified as quercetin 3-arabinoside. The other compounds were quantified as themselves. For each compound, calibration curves were performed, achieving high correlation coefficients (close to unit for all compounds).

### Statistics

All statistical analysis involving the experimental data were performed with R 2.1.1 for Linux, using the following packages: i) classical multivariate analysis library (mva); ii) main library of Venables and Ripley's (MASS); iii) Harrell miscellaneous (Hmisc); and iv) R-base packages [24]. Statistical analysis comprised the exploration of patterns and plausible data driven correlations between the phenolic profile and the studied factors: geographical location, year and cultivars. The first step of the analysis involved the calculation of the corresponding Pearson and Spearman correlation coefficients between the different phenolics to obtain the most important interactions between the quantified constituents [24, 26]. Furthermore, variance analysis was performed using the F-test statistical tests (α = 95%) to access the differences between phenolic compounds among the following factor data blocks: geographical location, production year and cultivar. The Tuckey multicomparison test was use to perform pair-wise comparisons among factor levels means. Pattern recognition analysis was performed using the principal component analysis technique (PCA), to access the correspondences between the different components of walnut leaf phenolics. Principal Components (PC) were analysed for their variance percentage, loadings and scores to access their significance. The Gabriel plot, using the principal components scores was performed to interpret patterns and discriminant walnut leaf phenolic components.
